# Dynamic Response Strategies: Accounting for Response Process Heterogeneity in IRTree Decision Nodes

**DOI:** 10.1007/s11336-023-09901-0

**Published:** 2023-02-06

**Authors:** Viola Merhof, Thorsten Meiser

**Affiliations:** Department of Psychology, University of Mannheim, L 13 15, 68161 Mannheim, Germany

**Keywords:** response styles, item response theory, multidimensional IRTree, item position effects

## Abstract

It is essential to control self-reported trait measurements for response style effects to ensure a valid interpretation of estimates. Traditional psychometric models facilitating such control consider item responses as the result of two kinds of response processes—based on the substantive trait, or based on response styles—and they assume that both of these processes have a constant influence across the items of a questionnaire. However, this homogeneity over items is not always given, for instance, if the respondents’ motivation declines throughout the questionnaire so that heuristic responding driven by response styles may gradually take over from cognitively effortful trait-based responding. The present study proposes two dynamic IRTree models, which account for systematic continuous changes and additional random fluctuations of response strategies, by defining item position-dependent trait and response style effects. Simulation analyses demonstrate that the proposed models accurately capture dynamic trajectories of response processes, as well as reliably detect the absence of dynamics, that is, identify constant response strategies. The continuous version of the dynamic model formalizes the underlying response strategies in a parsimonious way and is highly suitable as a cognitive model for investigating response strategy changes over items. The extended model with random fluctuations of strategies can adapt more closely to the item-specific effects of different response processes and thus is a well-fitting model with high flexibility. By using an empirical data set, the benefits of the proposed dynamic approaches over traditional IRTree models are illustrated under realistic conditions.

**Supplementary Information:**

The online version contains supplementary material available at 10.1007/s11336-023-09901-0.

Likert-type rating scales are widely used to assess personality, attitudes, or beliefs via self-reports. However, the validity of such trait measurements is threatened by response styles (RS)—tendencies to systematically respond to items on some basis other than what the items were designed to measure (Paulhus, 1991). RS comprise, for instance, preferences for the extreme categories (extreme RS; ERS) or the middle category of the scale (midpoint RS; MRS), irrespective of item content (for an overview, see Van Vaerenbergh & Thomas, 2013). Since RS can systematically bias estimates of substantive traits, resulting in inflated or underestimated individual scores, group means, and correlations of constructs, RS must be controlled for to ensure a valid interpretation of results (Alwin, 2007; Baumgartner & Steenkamp, 2001).

Various item response theory (IRT) approaches have been proposed that facilitate such control of RS effects under conditions in which the underlying response processes are homogeneous across persons and over items, thus assuming a stable response strategy over the course of a questionnaire (e.g., Böckenholt 2017; Bolt & Newton, 2011; Henninger & Meiser, 2020; Plieninger & Meiser, 2014; Wetzel & Carstensen, 2017). Extensions of such models can further account for some kind of heterogeneity of response processes over discrete conditions, either with a focus on latent classes of respondents (e.g., Kim & Bolt, 2021; Tijmstra et al., 2018; von Davier & Yamamoto, 2007) or with a focus on within-person changes across measurement occasions (e.g., Ames & Leventhal, 2021; Colombi et al., 2021; Weijters et al., 2010). Other approaches include unsystematic item-by-item fluctuations of response strategies within persons (e.g., Plieninger & Heck, 2018; Tijmstra & Bolsinova, in press; Ulitzsch et al., 2022). Neglected so far are *systematic* changes of response strategies *within* a measurement occasion and the associated heterogeneity regarding the manifestations of substantive traits and RS on the level of single items. We aim to close this research gap by modeling dynamic, item position-dependent influences of trait-based and RS-based response processes.

## Dynamic Trait and Response Style Effects

Whenever respondents are asked to provide subjective self-reports by responding to a Likert-type item, they are faced with the challenge of choosing one of several available categories. A prominent theory describing two competing response strategies to bring about such decisions is the conceptualization of Optimizing and Satisficing by Krosnick (1991). According to this framework, the currently applied response strategy depends on the respondents’ cognitive effort expended on item responses. On the one hand, giving accurate trait-based responses requires a substantial amount of effort, as four cognitive stages must be proceeded through. These are (1) comprehension of the item, (2) memory search for relevant information, (3) integration of pieces of information into a judgment, and (4) selecting a response category (Tourangeau et al., 2000). Responses derived from such processing are considered optimal, as they are accurate and strong indicators of the true trait levels. On the other hand, if respondents process some or all of the stages heuristically, item responses require less cognitive effort; they are not optimal, but still satisfactory from the respondents’ perspective (called satisficing responses). Unlike optimized, solely trait-based responses, such a satisficing response strategy is susceptible to the influence of RS (Aichholzer, 2013; Podsakoff et al., 2012). For instance, respondents may reach the global decision to agree or disagree with an item based on their trait level, but then do not consider the fine nuances between different options that indicate (dis)agreement. In such cases, individual category preferences determine the selection, so that extreme categories are chosen more often by respondents with high ERS levels, whereas midpoint responses are fostered by high levels of MRS. Metaphorically speaking, the decision vacuum left by a superficial instead of thorough trait-based selection is filled by RS-based processes. We, therefore, define *response strategy*, in the narrower sense, as a certain composition of trait-based response processes on the one side and heuristic processes related to one or several RS on the other side.

Whether predominantly trait-based or rather RS-based response strategies are used depends on the cognitive effort that respondents are able and willing to expend on the task, which in turn can be attributed to several properties of items and respondents (for an overview, see Podsakoff et al., 2012): For instance, low respondents’ abilities (e.g., low cognitive/verbal ability or education) and high task difficulty (e.g., a complex, abstract, or ambiguous item) can prevent the use of the optimizing, trait-based response strategy (Baumgartner & Steenkamp, 2001; Knowles & Condon, 1999; Krosnick, 1999; Messick, 1991; Podsakoff et al., 2003). Further, various properties of the measurement method (e.g., scale formats or contexts of data collection) were found to affect the degree of response style-related responding (DeCastellarnau, 2018; Van Vaerenbergh & Thomas, 2013). But even if a questionnaire is constructed and applied in a way that respondents are *able* to give optimized responses, insufficient motivation and fatigue can strengthen the RS influence and reduce the quality of responses (Galesic, 2006; Galesic & Bosnjak, 2009; Herzog & Bachman, 1981; Kahn & Cannell, 1957).

Whereas properties of the questionnaire and the response format can be considered fairly homogeneous and unsystematically varying across items, due to careful item construction and randomization, the respondents’ motivation for pursuing the high cognitive effort for optimizing responses may systematically change over time. In line with this, Krosnick (1991) states that “respondents are likely to satisfy whatever desires motivate them to participate just a short way into an interview, and they are likely to become increasingly fatigued, disinterested, impatient, and distracted as the interview progresses” (p. 214). Indeed, item responses are perceived as increasingly burdensome throughout a questionnaire (Galesic, 2006). In addition, long surveys and items presented in later parts of questionnaires reveal a lower data quality with more frequent omissions, dropouts, and response patterns indicating careless responding (Bowling et al., 2021a; Deutskens et al., 2004; Galesic & Bosnjak, 2009; Liu & Wronski, 2018; Marcus et al., 2007). Response times likewise indicate declining test-taking effort and a shift towards heuristic processing: They were found to be shorter for items presented toward the end of a questionnaire (Galesic & Bosnjak, 2009; Yan & Tourangeau, 2008), and such fast responses are associated with less motivation (Bowling et al., 2021b; Callegaro et al., 2009), satisficing responses in general (Andersen & Mayerl, 2017; Zhang & Conrad, 2014), and even more notably, responses that match the person-specific RS (Henninger & Plieninger, 2020). Thus, conditional on a substantial length of a questionnaire, respondents are likely to decrease their investment of cognitive capacity, and rather fall back to fast, heuristic processing. Such dynamic shifts in the response strategy result in a decreasing influence of the substantive trait, while the influence of RS increases over item position.

## Modeling Heterogeneity of Response Processes

The hypothesized dynamic influences of trait-based and RS-based processes reflect a within-person heterogeneity across the items of a questionnaire. There is a wide range of psychometric approaches accounting for heterogeneity in response processes with regard to RS, whereby the distinction between trait-based and RS-based processes has mainly been considered on the between-person level. For instance, mixture Rasch models (e.g., Austin et al., 2006; Gollwitzer et al., 2005, Meiser & Machunsky, 2008), mixture IRTree models (e.g., Khorramdel et al., 2019, Kim & Bolt, 2021), and a general mixture IRT model (Tijmstra et al., 2018) were proposed, which all can be used to identify latent classes of respondents who provide item responses based on different processes, such as responses influenced by response styles or not (i.e., solely trait-based responses). A limitation of such models is that the response process heterogeneity is strictly related to between-person effects so that possible class switches cannot be detected.

Other approaches allow to investigate the within-person stability of RS and to detect changes of respondents’ RS levels across discrete measurement occasions, like latent-state-trait models (Weijters et al., 2010; Wetzel et al., 2016), or longitudinal IRTree models (Ames & Leventhal, 2021). A stronger focus on heterogeneous response processes rather than on changes of RS levels per se is provided by hidden Markov models, in which respondents are assumed to hold one of several discrete latent states associated with a particular type of response process, and in which the assignment of respondents to states can change dynamically over measurement occasions (see Kelava & Brandt, 2019). For instance, Colombi et al. (2021) analyzed longitudinal item response data and defined two states, responding with or without the influence of RS, with part of the respondents modeled to freely switch between the two states. Similarly, Ulitzsch et al. (2022) proposed a response time-based mixture model, in which each response of a person is assumed to be stemming from either a careless or an attentive status. Furthermore, heterogeneity at the level of individual items was incorporated in some multi-process models, in which certain decisions during the selection of response categories are assumed to be based on one of several cognitively distinct processes (Plieninger & Heck, 2018; Thissen-Roe & Thissen, 2013; Tijmstra & Bolsinova, in press). For example, in the model by Plieninger and Heck (2018), affirmative responses can be either an expression of acquiescence RS or of trait-based agreement with the item content, though without accounting for systematically changing strategies.

Taken together, the past research linking RS modeling with heterogeneity of response processes within and between persons has mainly focused on: (1) discrete instead of continuous subpopulations or response states, (2) RS as an attribute that respondents may or may not have, instead of treating them as one of several processes that respondents can use to varying degrees, and (3) heterogeneity between measurement occasions or groups of items, instead of changes on the level of individual items.

In contrast, higher interest in continuous changes of response strategies within a measurement situation exists in item response modeling outside the RS literature. In the research field of performance decline, which describes a decreasing probability of correct responses for achievement items at the end of a test (for an overview, see List et al., 2017), the gradual process change model by Wollack and Cohen (2004) and Goegebeur et al. (2008) is a prominent model for generating and analyzing smooth changes in response strategies (e.g., Huang, 2020; Jin & Wang, 2014; Shao et al., 2016; Suh et al., 2012). In their approach, the response process of random guessing gradually takes over from trait-based problem-solving, and linear as well as curvilinear trajectories can be captured. In a later section of this article, we will account for shifts from effortful to more and more heuristic responses in a similar way, but instead of modeling random guessing for binary performance items, we model ordinal self-ratings and define heuristic responses as strongly influenced by RS.

Thereby, we aim to tackle the previous limitation of RS modeling, being that systematic within-person heterogeneity over the items of a questionnaire was not accounted for. Ignoring shifts in response processes is not only a potential problem for measuring and interpreting person and item parameters, as the dynamic changes themselves can also be the focus of interest: Measures of changes in trait and RS involvement can be used as a diagnostic tool to evaluate questionnaires with regard to the associated burden and required effort, and to compare, for example, subgroups of respondents (e.g., different age groups), subsets of items (e.g., positively and negatively worded items), or modes of data collection (e.g., online vs. lab). Furthermore, a formal model that describes dynamic response strategies can help to understand the interplay of cognitive processes that underlie item responses and to shed light on how respondents arrive at their judgments and decisions. Therefore, we not merely aim to control trait estimates for RS effects but also to provide a cognitive model accounting for dynamic response processes across items.

The remainder of this article is structured as follows: Firstly, traditional IRTree models are introduced. Then, a new dynamic IRTree model for continuously shifting influences of trait-based and RS-based processes is derived and evaluated by a first simulation study. Subsequently, a more flexible, non-continuous version of this model is introduced and likewise tested by a second simulation study. An empirical example is used to demonstrate the benefits of the dynamic approach under realistic conditions. Lastly, the results are interpreted and discussed in light of both basic and applied fields of research.

## IRTree Model Parameterizations of Traits and Response Styles

Multi-process IRTree models (Böckenholt, 2012; Böckenholt & Meiser, 2017; De Boeck & Partchev, 2012; Jeon & De Boeck, 2016) decompose response alternatives of rating scales into a sequence of binary pseudo-items, which represent the decisions assumed to be taken by the respondents during item responses. By assigning different latent traits to the pseudo-items, their effects on response selection can be separated. Typically, one pseudo-item represents the decision to agree vs. disagree with the item content, which is supposed to be made based on the substantive trait, whereas all further pseudo-items relate to RS-based responding, like the judgment to give extreme vs. non-extreme responses guided by ERS (e.g., Böckenholt, 2017; Khorramdel & von Davier, 2014; Plieninger & Meiser, 2014; Zettler et al., 2016).

### Unidimensional Node Parameterization

**Fig. 1:**
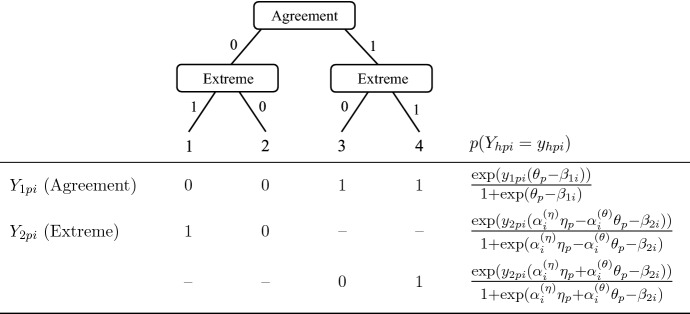
Tree diagram, definition of pseudo-items, and multidimensional node probabilities for responses to four-point Likert-type items. Due to the conditional definition of extreme responding, one of the two pseudo-item variants is missing by design for each ordinal category, as indicated by ’–’ . The item-specific laodings are constrained with $\alpha _i^{(\eta )} \ge 0$ and $\alpha _i^{(\theta )} \ge 0$.

In the following sections, we refer to items on a four-point Likert scale, and we decompose the ordinal item responses into decision nodes of broad agreement and fine-grained extreme responding based on the tree structure depicted in the upper part of of Fig. 1. The probability of the ordinal response $X_{pi} \in \{1,...,4\}$, representing the categories “strongly disagree”,“disagree”, “agree”, and “strongly agree” of person $p = 1,...,N$ to item $i = 1,...I$, is the product of the probabilities of responses to the two pseudo-items $Y_{hpi} \in \{0,1\}$ of agreement $(h=1)$ and extreme responding $(h=2)$. This model structure serves as an exemplary illustration for our new approach; dynamic response strategies can be easily adapted to differently structured trees and response formats with more or fewer ordinal categories (see Sect. 7 for an extension to five-point Likert-type items).

In the frequently applied Rasch IRTree, the two pseudo-items are each parameterized by a dichotomous Rasch model, with the agreement decision dependent on $\theta _p$, the person-specific substantive trait, and the extreme decision dependent on $\eta _p$, the person-specific ERS. Therefore, the ordinal category probability is obtained by:

1 $$\begin{aligned} p(X_{pi} = x_{pi}) = \left[ \frac{\text {exp}(y_{1pi}(\theta _p - \beta _{1i}))}{1 +\text {exp}(\theta _p - \beta _{1i})}\right] \left[ \frac{\text {exp}(y_{2pi}(\eta _p - \beta _{2i}))}{1 +\text {exp}(\eta _p - \beta _{2i})}\right] , \end{aligned}$$

where $\beta _{hi}$ denotes the difficulty of pseudo-item *h* of item *i*. Note that this definition of only one pseudo-item describing both extreme decision nodes reflects the assumption of identical decision-making processes for extreme agreement and disagreement (i.e., directional invariance of extreme responding, see Jeon & De Boeck, 2019).

### Multidimensional Node Parameterization

The traditional IRTree model with unidimensional nodes implies that each decision during the response selection is based on only one personal characteristic, either the substantive trait or a RS. However, as derived above, we rather assume that respondents consistently make a trait-based global decision to agree vs. disagree, but that the fine-grained decision in favor of the particular extreme or moderate category is guided by both trait-based and ERS-based processes, the composition of which is dependent on test-taking effort. In order to model this assumption, the extreme decision nodes can be extended by within-node multidimensionality (see Jeon & De Boeck, 2016; Meiser et al., 2019; von Davier & Khorramdel 2013) so that the respective pseudo-item responses are affected by both the trait and the ERS. In addition, the strict Rasch assumption of homogeneous item discrimination can be weakened by a 2PL parameterization with item-specific loadings of the person parameters so that influences of trait and ERS are not restricted to be constant throughout the questionnaire, but can vary depending on the item and its position within the questionnaire.

Figure 1 specifies this multidimensional 2PL parameterization of extreme responding, in which the item-specific response strategy is reflected by the loadings $\alpha _i^{(\theta )}$ and $\alpha _i^{(\eta )}$ of the substantive trait $\theta$ and ERS $\eta$, respectively. Further note that the extreme pseudo-item is split between the categories of disagreement and agreement, as proposed by Meiser et al. (2019): If the decision of agreement is answered affirmatively $(y_{1pi} = 1)$, extreme agreement is modeled to be more likely under high trait levels and high ERS levels. For disagreeing responses $(y_{1pi} = 0)$, in contrast, the trait loadings are set to be negative, so that high trait levels increase the probability of moderate (i.e., non-extreme) disagreement, whereas high ERS levels still make extreme disagreement more likely. Therefore, the ordinal category probability is obtained by

2 $$\begin{aligned} \begin{aligned} p(X_{pi} = x_{pi}) =&\left[ \frac{\text {exp}(y_{1pi}(\theta _p - \beta _{1i}))}{1 +\text {exp}(\theta _p - \beta _{1i})}\right] \left[ \frac{\text {exp}(y_{2pi}(\alpha _i^{(\eta )}\eta _p +\alpha _i^{(\theta )} \theta _p - \beta _{2i}))}{1 +\text {exp}(\alpha _i^{(\eta )} \eta _p + \alpha _i^{(\theta )} \theta _p - \beta _{2i})}\right] ^{y_{1pi}}\\&\left[ \frac{\text {exp}(y_{2pi}(\alpha _i^{(\eta )}\eta _p - \alpha _i^{(\theta )} \theta _p - \beta _{2i}))}{1 +\text {exp}(\alpha _i^{(\eta )}\eta _p - \alpha _i^{(\theta )} \theta _p - \beta _{2i})}\right] ^{1-y_{1pi}}, \end{aligned} \end{aligned}$$

with $$\alpha _i^{(\eta )} \ge 0 $$ and $$\alpha _i^{(\theta )} \ge 0$$.

## The Dynamic Response Strategy Model

**Fig. 2:**
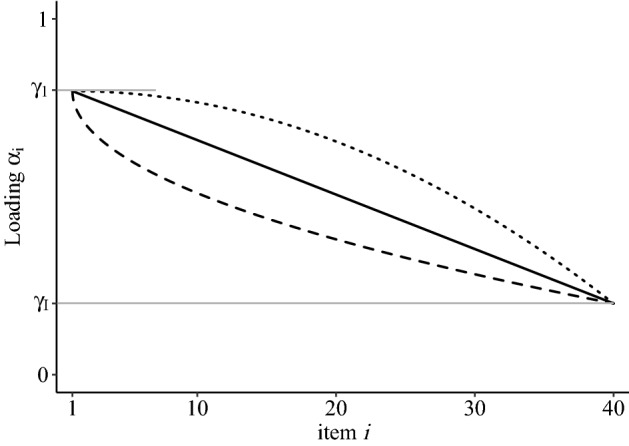
Relationship of loadings $\alpha _i$ and item position *i* for $I=40$ items with $\gamma _{1} = 0.8$, $\gamma _{I} = 0.2$ and $\lambda = 1$ (solid line), $\lambda = 2$ (dotted line), and $\lambda = 0.5$ (dashed line).

The novel dynamic response strategy model (DRSM) bases on the multidimensional IRTree parameterization and accounts for dynamic changes of response strategies over the course of the questionnaire by modeling the loadings of response processes as a function of item position. We use a modified form of the gradually changing function proposed by Wollack and Cohen (2004) and Goegebeur et al. (2008), which can capture linear as well as curvilinear relationships of a response process *p*, and is given by:

3 $$\begin{aligned} \alpha _i^{(p)} = \left( \gamma _{1}^{(p)}-\gamma _{I}^{(p)}\right) \; \left( 1- \left( \frac{i-1}{I-1}\right) ^{\lambda ^{(p)}}\right) + \gamma _{I}^{(p)}, \end{aligned}$$

with $\gamma _{1}^{(p)} \ge 0$, $\gamma _{I}^{(p)} \ge 0$, and $\lambda ^{(p)} \ge 0$. The parameters $\gamma _{1}^{(p)}$ and $\gamma _{I}^{(p)}$ are the loadings of process *p* of the first and last item, respectively. The actual dynamic change is captured by the slope, which is the difference between the last and first loadings ($\gamma _I^{(p)} - \gamma _1^{(p)}$). Therefore, a positive slope reflects a dynamically increasing influence, a negative slope reflects a decreasing influence, and a zero-slope trajectory reflects a non-dynamic, constant influence of the respective response process. In the further course of the article, we will frequently refer to the absolute slope, which accordingly describes the strength of the change, irrespective of the direction. The parameter $\lambda ^{(p)}$ determines the shape of the trajectory for process *p* over item position, which is linear for $\lambda ^{(p)} = 1$, and curvilinear otherwise (see Fig. 2).

The proposed DRSM for dynamic response strategies of extreme decisions can be derived by inserting a dynamic loading trajectory described by Eq. 3 into each the trait loadings and ERS loadings in Eq. 2. Thereby, the process loadings of the DRSM are defined to be nonnegative across all items, which is a frequently made assumption in IRT modeling (e.g., Jin & Wang, 2014; Kim & Bolt, 2021; Meiser et al., 2019). We consider this a reasonable constraint also for the loading trajectories, since variations in test-taking effort should result in a varying degree of trait and RS involvement, that is, in a varying size of the loadings, whereas a change toward negative loadings would rather imply a qualitatively different effect of such latent personal characteristics on response selection (e.g., high trait levels would then be associated with low instead of high response categories). Nonetheless, the DRSM could likewise be specified without this constraint, by allowing $\gamma _1^{(p)}$ and $\gamma _I^{(p)}$ to vary freely, in order to put the underlying assumption to the test. The consideration of negative loadings could additionally be a useful extension when some items are inverted with regard to the substantive trait,^1^ meaning that high trait levels are associated with endorsements of lower response categories. For such items, the DRSM could be adjusted by inverting the signs of the trait loadings in Eq. 2, so that the loadings of extreme agreement would be constrained negative and the loadings of extreme disagreement positive. The direction in which the trait influences the response selection (i.e., toward higher or lower categories) could thus be determined individually for each item, while the process loadings defined in Eq. 3 would reflect the strength with which a process is involved, without specifying the direction. Further note that the above parameterization of the DRSM refers to a fixed item order across respondents, as the same index *i* is used for the difficulty parameters and the response process loadings. An alternative approach would be the presentation of items in person-specific random order, for which the model can be adjusted accordingly, by defining item-dependent difficulties and position-dependent loadings.

Other reasonable modifications of the DRSM will be illustrated in this article, such as integrating dynamic influences of response processes not only into the two-dimensional decision nodes of extreme responding, but also into the unidimensional trait-based agreement decision (see Sect. 6). Further, IRTree models for response scales with more than four categories often include additional pseudo-items and additional RS, like decisions of moderate responding dependent on MRS, and such can likewise be modeled by the DRSM (see Sect. 7). Moreover, the DRSM as described above considers item position as the only predictor of response process loadings, thus implying a continuous response strategy with monotonically changing loadings. This is a theoretical model with an explicit focus on item position as *one* of possibly several factors influencing the impact of trait-based and RS-based processes within each item. However, in the context of the second simulation study, we derive a flexible extension of the DRSM, which still accounts for dynamic loading trajectories, but at the same time can capture further (random) item-specific variation of loadings.

### Evaluating the Dynamic Response Strategy Model

Two simulation studies were conducted to systematically evaluate the proposed dynamic modeling approaches (DRSM and an extended version), and to provide answers to the following questions: Firstly, is the DRSM an appropriate cognitive explanatory model that reliably detects and quantifies dynamic influences of response processes in the data? Secondly, is the DRSM a beneficial psychometric measurement model that creates added value for the analysis of item response data over existing models? Both questions were investigated under ideal conditions, in which the data-generating model followed a continuous, model-implied response strategy (Sect. 5), and under more realistic conditions, in which additional random variation was added (Sect. 6). The proposed dynamic models were evaluated in relative comparison to IRTree models representing reasonable alternatives.

## Simulation Study 1

In the first simulation study, we addressed dynamic and non-dynamic continuous response strategies, meaning that the trait and ERS loadings were constrained by continuous trajectories so that influences of response processes were only depend on the position of items within the questionnaire. For such scenarios, we examined the accuracy of the DRSM in recovering dynamic response strategies (i.e., sensitivity to detect changes in the impact of trait and ERS), as well as the risk of false-positive dynamics, which is finding such an effect if it is not present in the data (i.e., specificity of unveiling dynamic changes only in cases where they do exist). Further, we investigated the recovery of person and item parameters and the out-of-sample model fit in comparison with alternative IRTree models.

### Models of Continuous Response Strategies

**Table 1:** Loading constraints of models with continuous response strategies used in simulation study 1.

Model	Trait loading $\alpha _i^{(\theta )}$	ERS loading $\alpha _i^{(\eta )}$
DRSM	$\; (\gamma _{1}^{(\theta )}-\gamma _{I}^{(\theta )}) \; \left( 1- \left( \frac{i-1}{I-1}\right) ^{\lambda ^{(\theta )}}\right) + \gamma _{I}^{(\theta )}\;$	$\;(\gamma _{1}^{(\eta )}-\gamma _{I}^{(\eta )}) \; \left( 1- \left( \frac{i-1}{I-1}\right) ^{\lambda ^{(\eta )}}\right) + \gamma _{I}^{(\eta )}\;$
Static	$\alpha ^{(\theta )}$	$\alpha ^{(\eta )}$
ERS	0	1
Ordinal	1	0

The simulation study covered item response data under the assumption of continuous response strategies, and all applied models can be derived from the general model structure described in Fig. 1 and Eq. 2. The parameterization of the agreement pseudo-item was equal across models and corresponded to a solely trait-dependent unidimensional Rasch model. The two-dimensional definition of the extreme pseudo-item with item-specific loadings of trait and ERS served as a superordinate framework, from which we derived unidimensional as well as two-dimensional special cases, as defined in Table 1.

Under the DRSM, the item-specific loadings were determined by the trait and ERS trajectories, which comprise the three parameters $\gamma _1$, $\gamma _I$, and $\lambda$ each. The static model excludes such a change over items, but rather assumes a single constant loading for each of the two processes. Besides those models with two-dimensional response strategies, also unidimensional models were derived. Their extreme decision nodes were Rasch parameterized and included only one of the person parameters with constant loading 1, that is the ERS $\eta$ for the ERS model and the substantive trait $\theta$ for the ordinal model. The loadings of the respective other parameter were set to 0. The ERS model is equivalent to the traditional IRTree described by Eq. 1 and the ordinal model corresponds to its counterpart as was proposed by Kim and Bolt (2021).

### Data Generation

Using R (R Core Team, 2020), item response data were generated according to the four IRTree models with continuous response strategies described above. In the DRSM, the trait and ERS loading trajectories were systematically varied to cover a wide range of plausible dynamic response strategies (resulting in six model variants). We only simulated decreasing trait loadings and increasing ERS loadings, as these correspond to our theoretical consideration, and analogous models can be specified for opposite trajectories. The trait loading trajectories were generated with $(\gamma _{1}; \gamma _{I})$ set to (0.8; 0.2), (0.7; 0.3), (0.6; 0.4), and (0.5; 0.5), so that the absolute slopes were of size 0.6, 0.4, 0.2, and 0.0. Likewise, the ERS loading trajectories were set to (0.2; 0.8), (0.3; 0.7), (0.4; 0.6), and (0.5; 0.5).^2^ We combined trait and ERS trajectories with different absolute slopes so that the response strategy change was either large (i.e., one process changed by 0.6, the other by 0.4), medium (0.4 and 0.2), or small (0.2 and 0.0). For each generated data set, both trajectories were generated with the same value of $\lambda$, set to 2 or 0.5, which we considered as reasonable values for a positively or negatively accelerated dynamic change, respectively. For data generation with the static model, two model variants were defined, which are the constant trait and ERS loadings $(\alpha ^{(\theta )}; \alpha ^{(\eta )})$ set to (0.3; 0.7) and (0.7; 0.3). The two unidimensional models have fixed trait and ERS loadings and thus do not require to specify additional parameters.

For all model variants, 100 replications were conducted each for two sample sizes *N*, set to 500 and 1000, and with the two questionnaire lengths *I*, set to 20 and 40. Each data set consisted of binary responses to the two pseudo-items of agreement and extreme responding under a certain model variant and was generated as follows: Firstly, the person parameters, that are *N* trait levels $\theta _p$ and *N* ERS levels $\eta _p$, were generated to be uncorrelated and sampled from independent standard normal distributions. Likewise, 2*I* pseudo-item difficulties $\beta _{hi}$ were randomly drawn from a standard normal distribution. Then, person and item parameters were inserted into the respective equation of the model variant with its item-specific trait and ERS loadings. Lastly, for each person and each item, binary responses to the pseudo-items were randomly sampled according to the model-implied probabilities.

### Model Estimation and Analysis

Each data generation step was followed by a model estimation step, in which all four models with continuous response strategy changes (see Table 1) were applied to the respective data set. In addition, also a 2PL model with freely estimated item-specific trait and ERS loadings was fitted (the agreement node was Rasch parameterized as in the other models). The 2PL model is not specifically targeted at continuous response strategies, but as all previously described continuous models are nested within it, it could be a flexible, universal alternative.

Bayesian parameter estimation was performed using the No-U-Turn Sampler (Hoffman & Gelman, 2014), a Markov chain Monte Carlo algorithm implemented in the software program Stan (Carpenter et al., 2017). R served as the interface to Stan along with the package CmdStanR (Gabry & Cešnovar, 2021). Four chains were run with each 1000 iterations and a warmup of 500 iterations. All estimated models reached convergence, indicated by values of the potential scale reduction factor $\widehat{R}$ less than 1.05. Note that all point estimates reported in the following sections are the expected a posteriori (EAP) estimates.

Priors were chosen according to recommendations in the Bayesian IRT literature (e.g., Luo & Jiao, 2018; Stan Development Team, 2020). The priors for $\theta _p$ and $\eta _p$ were set to standard normal distributions, and a normally distributed hierarchical prior was applied to the item difficulties $\beta _ {hi}$ with a *Cauchy*(0, 5) hyperprior for the mean and nonnegative *Cauchy*(0, 5) for the standard deviation. Weakly informative *LogNormal*(0, 2) priors were placed on (1) $\gamma _{1}$ and $\gamma _{I}$ of the DRSM trajectories, (2) the constant loadings of the static model, and (3) the item-specific trait and ERS loadings of the 2PL model ($\alpha _i^{(\theta )}$, $\alpha _i^{(\eta )}$). This ensured the convergence of the models even under conditions in which a response process did not contribute to the data generation, but needed to be estimated by the model (e.g., estimating the influence of the ERS for ordinal data). The shape $\lambda$ of the DRSM was defined in the interval [0.25, 4] and given a $LogNormal(-0.5, 1)$ prior. Again, this prior was chosen to ensure convergence, as only weak information is available to estimate $\lambda$ if the difference of $\gamma _{1}$ and $\gamma _{I}$ is small. In an extreme case of a zero-slope trajectory, $\lambda$ can take any value without having an effect on the loading trajectory. Further, the interval boundaries were chosen to account for the fact that $\lambda$ and $\frac{1}{\lambda }$ have symmetrical effects on the curvature of the trajectory, though in the opposite directions (see Fig. 2) and assured that the estimated shapes were within the range of plausible trajectories that we considered to be continuously and not abruptly changing.

### Results

**Fig. 3:**
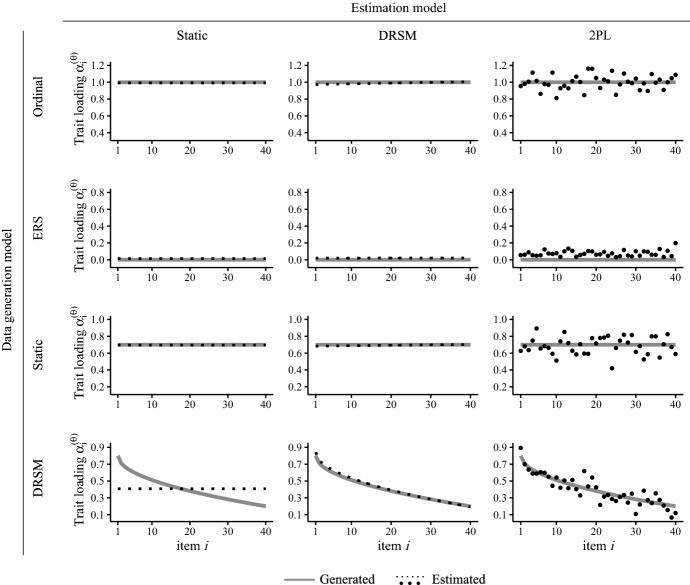
Trait loading estimates of the static model, DRSM, and 2PL model to exemplary data sets generated by the four models of continuous response strategies used in simulation study 1. Trait loadings of the ordinal and ERS model are not shown, as they are not estimated, but fixed at 1 and 0, respectively.

The DRSM and the four alternative models considered in the simulation study only differed in their constraints regarding the loadings of response processes. To give an overview of how the models behaved when fitted to item response data generated under such constraints, Fig. 3 illustrates the trait loading estimates provided by the different models for exemplary data sets. Even though these examples cannot summarize the entirety of simulations, beneficial characteristics of the DRSM in comparison with alternative models become clear, which we will elaborate on in the following.^3^

#### Sensitivity: Estimation of Dynamic Trajectories

The first aim of the simulation was to examine the sensitivity of the DRSM, and accordingly, to answer the question of whether it is suitable for detecting and quantifying dynamic trajectories of trait and ERS loadings. Therefore, we evaluated (1) the recovery of the slope ($\gamma _I - \gamma _ 1$), which is probably the most informative dynamic measure, as it quantifies the change over the course of the questionnaire, (2) the recovery of the shape $\lambda$, and (3) the precision of estimates of the trajectory parameters $\gamma _1$, $\gamma _I$, and $\lambda$.

**Fig. 4:**
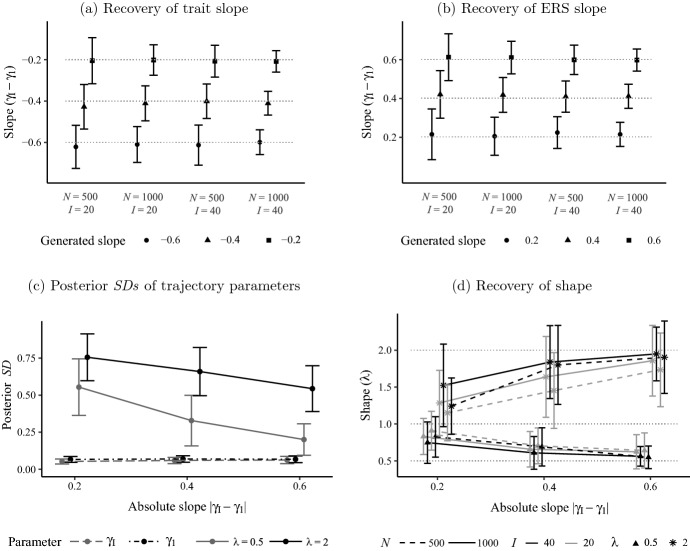
Estimates and precision of trajectory parameters by the DRSM for continuous dynamic data in simulation study 1. Error bars represent the *SDs* of estimates across simulation replications.

Figure 4a, b summarizes the slope estimates of trait and ERS loadings by the DRSM and reveals a good recovery of both trajectories. Irrespective of sample size and questionnaire length, the means across simulation replications closely matched the respective true generated values. The good recovery of slopes is in line with consistently small posterior *SDs* of $\gamma _1$ and $\gamma _I$ (two bottom lines in Fig. 4c), meaning that they were estimated quite precisely. In contrast, $\lambda$ estimates had high uncertainty and the posterior *SDs* were a multiple of those of the other two parameters. Note that the difference in posterior *SDs* between $\lambda$ set to 0.5 and 2, as apparent in Fig. 4c, stems from the nonlinear relationship of $\lambda$ and trajectory curvature, since the smaller $\lambda$ is, the large the change in the degree of curvature induced by slight changes in the parameter. In general, all three trajectory parameters were estimated more precisely the larger the size of the data set, determined by *N* and *I* (see Table A1). Moreover, the larger the absolute slope, the higher the precision of $\lambda$ estimates, whereas estimates of $\gamma _1$ and $\gamma _I$ were not affected by the slope.

The link between slope and shape was already discussed with regard to the informative prior on $\lambda$, which was imposed to assure convergence in case of flat trajectories. Accordingly, the $\lambda$ estimates of trajectories with small absolute slopes were highly influenced by the prior, which moved the parameter toward the value 1 (see Fig. 4d). The steeper the trajectories, the more information regarding the shape was provided by the data, the prior had less influence, and the $\lambda$ estimates moved closer to the true values of 2 and 0.5, respectively. However, even for large slopes, the uncertainty of $\lambda$ estimates was high so that EAP estimates should not be interpreted in a substantial manner without considering the uncertainty (e.g., the rough classification as positively or negatively accelerated trajectory is possible if the credible interval (CI) does not contain the value 1). Altogether, the analysis of sensitivity demonstrated that the DRSM is an appropriate cognitive model for investigating dynamic response strategies; it was highly suitable to detect systematic changes of response process influences, to assess the magnitude of such changes with high precision, and to roughly inform about the shape of the trajectory, if the DRSM itself was used to generate the data.

#### Specificity: Estimation of Non-dynamic Trajectories

Furthermore, also the specificity was to be examined, that is, whether the model accurately detected the absence of dynamic changes. To answer this question, we evaluated simulation conditions in which the DRSM was fitted to non-dynamic data generated by the unidimensional models and the static model, which all have zero-slope trajectories. Indeed, the estimated slopes by the DRSM were all very close to 0 and had low variance (see Table A2), demonstrating that the model consistently detected the absence of dynamic changes (also see the illustration of estimated trajectories in exemplary zero-slope data sets in Fig. 3). The more parsimonious models were successfully mimicked, meaning that the estimated parameters by the DRSM reflected the restrictions of models nested within it. Therefore, the DRSM is a suitable cognitive model also for data with non-dynamic response strategies.

#### Parameter Recovery

**Fig. 5:**
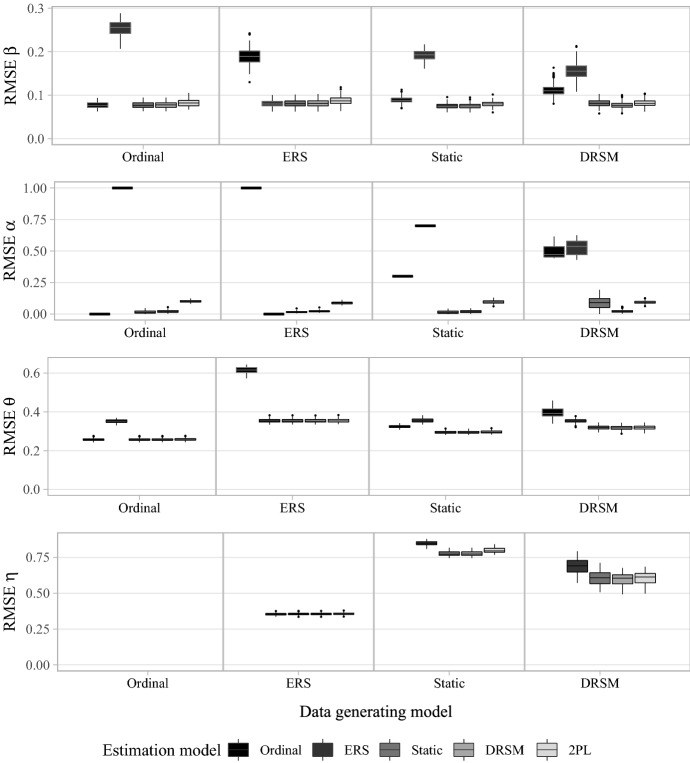
RMSEs of estimated person and item parameters for continuous data in simulation study 1. The boxplots summarize the results for the simulation condition with $N =1000$ and $I=40$. For data generation with the static model, only the condition with $\alpha _i^{(\theta )} = 0.7$; $\alpha _i^{(\eta )} = 0.3$ is shown. RMSEs of $\eta$ estimates for data generated with or estimated by the ordinal model are missing, as the model does not incorporate an ERS influence.

Besides investigating the adequacy of the DRSM to accurately describe different response strategies, we aimed to examine its quality as a psychometric model. To this end, the recovery of person parameters ($\theta _p$ and $\eta _p$) and item parameters ($\beta _{hi}$ and $\alpha _{i}$) was compared across models, measured by root mean square error (RMSE). There were only minor differences in parameter recovery between conditions with different sample sizes or questionnaire lengths, except that the overall levels of RMSEs were smaller, the larger the data set (see Fig. A1). The results for conditions with $N =1000$ and $I=40$ are illustrated in Fig. 5. In general, the models with two-dimensional extreme decision nodes (static model, DRSM, and 2PL model) yielded considerably smaller errors compared to the unidimensional models (ordinal and ERS). The unidimensional models showed good parameter recovery only for data sets generated by the respective model itself but performed poorly for data generation with all other models.

The two-dimensional models, in contrast, were nearly equally well suited to recover the parameters of all kinds of data sets, with the exception of the response process loadings. Here, the 2PL model revealed comparably large errors, which is in line with the finding that its freely estimated loadings do not perfectly mimic the underlying dynamic or non-dynamic continuous trajectories, but rather scatter around them (see Fig. 3). The static model and DRSM recovered all parameters of models nested within them well, though unsurprisingly, the static model showed larger errors for the loadings of the DRSM. This shows that the DRSM was the model with the overall best parameter recovery and that it did not pose a risk of increased errors in case of misspecifications, but successfully mimicked lower-parameterized models.

#### Model Fit

The five models were further compared with regard to their fit, for which we calculated the out-of-sample prediction accuracy by an approximation of leave-one-out cross-validation (LOO; Vehtari et al., 2017). LOO is a fully Bayesian information criterion, which has been shown to outperform alternative methods like Akaike’s information criterion (AIC; Akaike, 1974) or the deviance information criterion (DIC; Spiegelhalter et al., 2002) in IRT model selection (Luo & Al-Harbi, 2017). Table 2 lists the average LOO information criterion values (small values indicate better fit), as well as the proportion of simulation replications in which the respective model was the best one in predicting the data. Across all conditions, the respective data-generating model itself provided the best out-of-sample fit in the majority of replications and entailed the smallest average LOO values. The DRSM was almost always selected as the best-fitting model for data sets with dynamic response strategies of medium or large size. For small response strategy changes (i.e., one process changed with absolute slope 0.2, the other was constant with slope 0.0), the DRSM was still the best-fitting model, though also the static model often predicted the data well. This condition of small dynamic changes was the only one in which we found substantial differences across sample sizes and questionnaire lengths: For $N=500$ and $I=20$, the DRSM itself was selected in only 50 % of replications, whereas it was selected in 92 % for $N=1000$ and $I=40$. Thus, only if limited data was available, the zero-slope trajectories of the static model gave a good approximation of dynamic loadings. The larger the data set, the more evident the advantage of the more complex DRSM and its additionally estimated parameters was. For data sets generated by the unidimensional models, not only the respective model itself, but also the static model and DRSM predicted the data well, as can be seen by average LOO values quite close to the values of the data-generating model. This can be explained by the fact that both two-dimensional models closely mimicked the lower-parameterized models and did not overfit despite their greater flexibility (see the good recovery of person and item parameters in Figs. 5 and 3). The same holds true for static data, which was mostly best predicted by the static model itself, but for which the DRSM also revealed small LOO values. In contrast, the 2PL model was hardly ever selected as the best-fitting model, and the average LOO values were considerably larger than those of the DRSM. The analysis of the parameter recovery suggests that these indicators of overfitting and reduced generalizability of the 2PL model are mainly due to the comparably poor recovery of loading estimates. The DRSM thus offered the best compromise between flexibility on the one hand, and generalizability on the other hand, if dynamic or non-dynamic continuous response strategies are present in the data. The model not only proved to be a valuable cognitive model of response strategies with high sensitivity (i.e., dynamic changes of response process influences over items are reliably captured) and high specificity (i.e., dynamic changes are not falsely revealed)—it is further a beneficial psychometric measurement model, which provides added value in terms of parameter recovery and models selection.

**Table 2:** Model comparisons by LOO out-of-sample prediction accuracy for continuous data in simulation study 1.

Data generation		Average LOO information criterion					Proportion of replications in favor of				
Model	Abs. slopes	ORD	ERS	Static	DRSM	2PL	ORD	ERS	Static	DRSM	2PL
ORD	0/0	**87,419**	92,860	87,422	87,423	87,492	**0.68**	0	0.22	0.10	0
ERS	0/0	96,649	**88,135**	88,137	88,139	88,212	0	**0.58**	0.28	0.14	0
Static	0/0	92,071	91,416	**89,751**	89,754	89,853	0	0	**0.74**	0.26	0
DRSM	0.2/0.0	91,885	91,465	90,036	**90,019**	90,125	0	0	0.25	**0.75**	0
	0.4/0.2	92,053	91,755	90,264	**90,163**	90,266	0	0	0.01	**0.99**	0
	0.6/0.4	91,872	91,665	90,107	**89,841**	89,942	0	0	0	**1.00**	0

The LOO values and proportions in bold indicate the overall best-fitting model in the respective data generation condition.

The average LOO information criterion values include the replications with $N = 1000$ and $I = 40$. The other conditions yielded comparable patterns.

## Simulation Study 2

In order to further investigate the benefits of the DRSM under real-world conditions, the second simulation study addressed non-continuous dynamic response strategies. They are characterized by a general trend of loadings over the course of the questionnaire, but, in contrast to continuous strategies, allow for item-specific deviations from the trajectories. Such loading patterns are probably more frequently encountered in empirical data than strictly continuous trajectories, because even though previous research indicated that item position is a crucial factor influencing the impact of response processes, additional item-specific variation of loadings could arise; for instance, due to the items’ levels of abstractness or complexity (e.g., positively vs. negatively worded items, grammatical or linguistic complexity). Such additional variance between items does hardly affect the overall dynamic response strategy change across item positions and, as a result, should not limit the validity of the DRSM as a cognitive model. Thus, the first aim of the simulation study is to put this assumption to the test and to analyze the performance of the DRSM in detecting dynamic changes in the presence of additional random variation of loadings. However, even if general changes in the response behavior can be detected, the DRSM would simplify the true, underlying data-generating processes. Therefore, we propose a more flexible extension, the F-DRSM, which can capture the hypothesized non-continuous dynamic response strategies in addition to systematic underlying trajectories. Thus, the second aim of the simulation study is to evaluate the F-DRSM and to examine its psychometric properties.

### The Flexible Dynamic Response Strategy Model

The F-DRSM can be seen as a combination of the DRSM and 2PL model: The item-specific loadings are the sum of a systematic component, which is defined by a continuous trajectory, and unsystematic, random noise. Fixing this unsystematic component for each item to 0 would yield the DRSM, whereas omitting the systematic trajectory component would result in the 2PL model. In the F-DRSM, the random noise is assumed to stem from a common normal distribution across all items, in which the mean is fixed to zero, and the standard deviation indicates the strength of the deviation from the trajectory. The F-DRSM loadings of a response process *p* are defined by:

4 $$\begin{aligned} \alpha _i^{(p)}&\sim Normal(\mu _i^{(p)}, \sigma ^{(p)}),\nonumber \\ \mu _i^{(p)}&= (\gamma _{1}^{(p)}-\gamma _{I}^{(p)}) \; \left( 1- \left( \frac{i-1}{I-1}\right) ^{\lambda ^{(p)}}\right) + \gamma _{I}^{(p)},\nonumber \\ \sigma ^{(p)}&\sim Cauchy(0,5). \end{aligned}$$

The F-DRSM provides estimates for (1) the item-specific loadings $\alpha _i^{(p)}$, (2) the underlying dynamic trajectory, and (3) the standard deviation $\sigma ^{(p)}$, with which the loadings scatter around the trajectory. Thereby, the item-specific loadings are allowed to deviate from the respective values predicted by the common trajectory ($\mu _i^{(p)}$) but are at the same time shrunken to this mean. The trajectory can thus be seen as a prior for the free loadings, which is not fixed but estimated from the data. Whether this prior is rather informative or uninformative depends on the data: If almost all item-specific loadings of a process closely fit a trajectory, the remaining loadings are strongly shrunken to this trajectory. In contrast, if the data suggest that the loadings largely scatter and do not form a trajectory, the individual loading estimates are hardly affected by the trajectory estimate, and the F-DRSM converges to the standard 2PL model. As the extent of unsystematic variation of loadings does not have to be determined a priori, but emerges as an estimate from the model, hypotheses regarding the strength of the continuous trend can be tested. Moreover, the variance of loadings does not have to remain unsystematic but can be explained by further predictors.

### Data Generation and Model Estimation

All generated data sets of the second simulation study contained item responses of $N=1000$ respondents to $I=40$ items on a four-point scale under the model assumptions of the F-DRSM. In contrast to the first simulation study, not only the two-dimensional IRTree nodes of extreme responding but also the unidimensional agreement node was given a 2PL parameterization with item-specific trait loadings, so that the ordinal category probability was given by:

5 $$\begin{aligned} \begin{aligned} p(X_{pi} = x_{pi}) =&\left[ \frac{\text {exp}(y_{1pi}(\alpha _{1i}^{(\theta )} \theta _p - \beta _{1i}))}{1 +\text {exp}(\alpha _{1i}^{(\theta )} \theta _p - \beta _{1i})}\right] \left[ \frac{\text {exp}(y_{2pi}(\alpha _i^{(\eta )}\eta _p +\alpha _{2i}^{(\theta )} \theta _p - \beta _{2i}))}{1 +\text {exp}(\alpha _i^{(\eta )} \eta _p + \alpha _{2i}^{(\theta )} \theta _p - \beta _{2i})}\right] ^{y_{1pi}}\\&\left[ \frac{\text {exp}(y_{2pi}(\alpha _i^{(\eta )}\eta _p - \alpha _{2i}^{(\theta )} \theta _p - \beta _{2i}))}{1 +\text {exp}(\alpha _i^{(\eta )}\eta _p - \alpha _{2i}^{(\theta )} \theta _p - \beta _{2i})}\right] ^{1-y_{1pi}}. \end{aligned} \end{aligned}$$

**Fig. 6:**
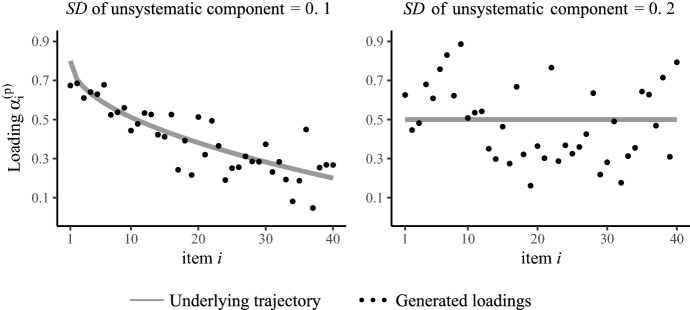
Examples of randomly generated loadings under the F-DRSM.

Thus, three noisy trajectories were defined, that are the trait loadings of agreement ($\alpha _{1i}^{(\theta )}$), the trait loadings of extreme responding ($\alpha _{2i}^{(\theta )}$), and the ERS loadings of extreme responding ($\alpha _i^{(\eta )}$). The absolute slopes of these three trajectories were varied (0.0, 0.2, 0.4, 0.6), whereby all trait trajectories decreased over items and the ERS trajectories increased. The standard deviation of the unsystematic noise was set to either 0.1 or 0.2 for all trajectories. All further settings were chosen as in the first study. Examples of the resulting non-continuous dynamic loadings for different underlying trajectories are given in Fig. 6. We generated 100 data sets for each model variant and fitted the DRSM, the F-DRSM, and the 2PL model, using the same Bayesian parameter estimation and priors as in the first study.

### Results

#### Slope Estimates

To evaluate the utility of the two dynamic models as cognitive models for describing systematic changes in response strategies, we analyzed the recovery of the slopes of the three response process trajectories. In addition, also the slope recovery of the 2PL model was investigated, which does not provide estimates of trajectories inherently, so that we fitted dynamic functions through the freely estimated loadings in a post hoc analysis. In contrast to the F-DRSM, the estimation of the loadings in the 2PL model is independent of the estimation of the trajectories.

Notably, the DRSM, F-DRSM, and 2PL model recovered the slopes equally well, irrespective of the size of the slope and irrespective of the *SD* of the unsystematic component, demonstrating that dynamic as well as non-dynamic response processes were successfully detected (see Table A3 for the comparison of slope estimates by the three models). Moreover, the data set-specific slope estimates of DRSM, F-DRSM, and 2PL model hardly differed from each other and were highly correlated, which underlines that the models drew almost identical conclusions regarding the response strategy changes. This suggests that, for the sole sake of determining the size of strategy changes, there is no practical difference between (1) taking a continuous dynamic trajectory as a direct estimate of individual loadings, (2) using it as a prior that shrinks the loadings, or (3) fitting the trajectory after estimating the loadings freely. However, the models largely differed in the uncertainty with which the slopes were estimated, as the DRSM yielded considerably higher precision of trajectory parameter estimates and had the smallest posterior *SDs* of slopes. Thus, the DRSM enables more specific conclusions to be drawn about the extent of response strategy changes (e.g., whether the change is different from 0, or whether there are differences between groups of items or persons), so that it should be preferred over the other models for response behavior analyses.

#### Model Fit and Parameter Recovery

Furthermore, the three models also differed in their suitability as psychometric models: Model comparisons by LOO out-of-sample prediction accuracy clearly showed the benefits of the F-DRSM, which provided the smallest LOO values and was chosen as the best-fitting model in all replications (see Table 3). Even under conditions with large unsystematic *SD* of 0.2, in which the item-specific loadings largely scatter so that trajectories are hardly recognizable (see Fig. 6), the F-DRSM was advantageous over the unrestricted estimation by the 2PL model. Unsurprisingly, the DRSM could not predict the data well, since it cannot properly capture the non-continuous patterns of loadings.

**Fig. 7:**
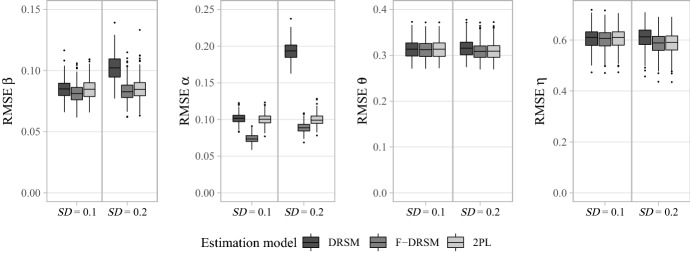
RMSEs of estimated person and item parameters for non-continuous data in simulation study 2.

Likewise, the analysis of parameter recovery demonstrated the superiority of the F-DRSM, which yielded the smallest errors across person and item parameters (see Fig. 7). As was the case in the first simulation study, also the 2PL model provided a good recovery, except for slightly higher RMSEs of loadings. The DRSM showed comparably high errors for the item parameters but still recovered the person-specific substantive trait levels as accurately as the higher-parameterized models. This suggests that the model adjusted the item difficulties in a way that they counteracted the deviations of individual loadings from a continuous trajectory. In line with this, the smaller the unsystematic *SD*, the smaller the disadvantage of the DRSM compared to the more flexible models.

**Table 3:** Model comparisons by LOO out-of-sample prediction accuracy for non-continuous data in simulation study 2.

Data generation		Average LOO information criterion			Proportion of replications in favor of		
Abs. slope	*SD*	DRSM	F-DRSM	2PL	DRSM	F-DRSM	2PL
0.0	0.1	90,230	**90,140**	90,210	0	**1.00**	0
	0.2	90,365	**89,836**	89,864	0	**1.00**	0
0.2	0.1	89,969	**89,883**	89,952	0	**1.00**	0
	0.2	90,509	**89,987**	90,015	0	**1.00**	0
0.4	0.1	89,926	**89,840**	89,904	0	**1.00**	0
	0.2	89,814	**89,328**	89,354	0	**1.00**	0
0.6	0.1	89,605	**89,521**	89,582	0	**1.00**	0
	0.2	89,638	**89,162**	89,184	0	**1.00**	0

The LOO values and proportions in bold indicate the overall best-fitting model in the respective data generation condition.

Overall, the second simulation study clearly showed the benefits of modeling dynamic response strategies under real-world conditions and revealed that both the DRSM and the F-DRSM are models with high utility, though for different kinds of research goals. On the one hand, the DRSM accurately reflected the magnitude of response strategy changes over the items of a questionnaire, and although the two alternative models produced almost identical results at the level of point estimates, the DRSM had the advantage of more precise estimates. Therefore, we recommend using the DRSM as a cognitive explanatory model if the goal is to analyze data sets with a focus on investigating the respondents’ behavior. Further, the recovery of person-specific trait levels was hardly affected by model choice, making the DRSM a parsimonious and convenient model for controlling substantive trait estimates for dynamic RS effects. Nevertheless, the DRSM inevitably simplifies the true loading patterns whenever strategy changes are not perfectly continuous. Therefore, the more flexible F-DRSM is the preferable model for investigating influences of response processes on the level of individual items, as it can accurately capture random fluctuations of response strategies in addition to the underlying continuous trend. Further, it was even better suited than a 2PL model in terms of model selection and parameter recovery, so the F-DRSM has proven to be a beneficial psychometric model for the analysis of item response data under influence of response style effects.

## Empirical Application

**Fig. 8:**
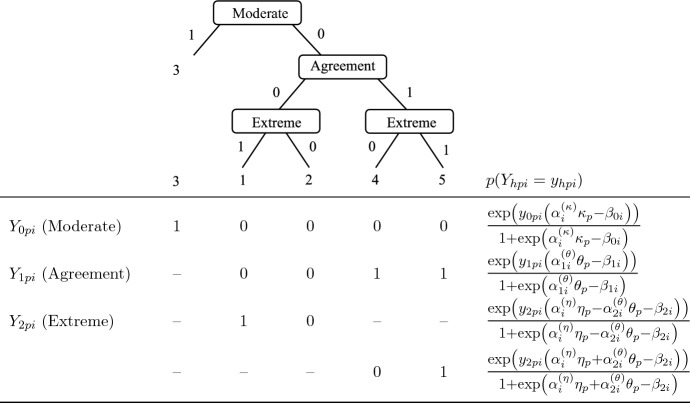
Tree diagram, definition of pseudo-items, and node probabilities for responses to five-point Likert-type items. Pseudo-items that are missing by design are marked with ’–’.

To demonstrate the advantages of modeling response strategy changes in real data, we applied the proposed dynamic models as well as the other models used in the simulation studies to an empirical data set taken from Johnson (2014). The data consists of item responses to the IPIP-NEO-120 with 120 items on five broad personality domains (Neuroticism, Extraversion, Conscientiousness, Agreeableness, and Openness to Experience), and we randomly selected 1000 participants of the large online sample from the American population with complete data. The response scale of the IPIP-NEO-120 comprises five rating categories, which is why we extended the tree structure of Fig. 1 by an additional node of moderate vs. non-moderate responding (e.g., Böckenholt, 2012; Böckenholt & Meiser, 2017; Khorramdel & von Davier, 2014). The extended tree of five-point rating scales is depicted in Fig. 8.

The decision of moderate responding was modeled to be dependent on the respondent’s midpoint RS (MRS) $\kappa _p$, weighted with an item-specific loading $\alpha _{i}^{(\kappa )}$, and on the item difficulty $\beta _{0i}$ of the additional pseudo-item ($h = 0$). As was done in the second simulation study, the trait loadings of the agreement node ($\alpha _{1i}^{(\theta )}$) were not fixed but estimated so that our theoretical assumption of dynamically changing response strategies predominantly occurring in fine-grained decisions could be empirically tested. Thus, all four response processes (MRS-based moderate responding, trait-based agreement, trait-based extreme responding, and ERS-based extreme responding) had independent item-specific loadings, which were either fixed (ordinal and ERS model), or estimated according to the model-specific constraints. Further, we let the variances of person parameters be estimated unless a standard normal prior was needed for model identification (i.e., one of the five traits and both RS for the static model and DRSM; all traits and both RS for the F-DRSM and 2PL model). We used the same Bayesian model estimation as in the simulation studies, with four chains, 500 warmup iterations, and 1000 post-warmup iterations to assure convergence (all $\widehat{R}<1.05$).

**Fig. 9:**
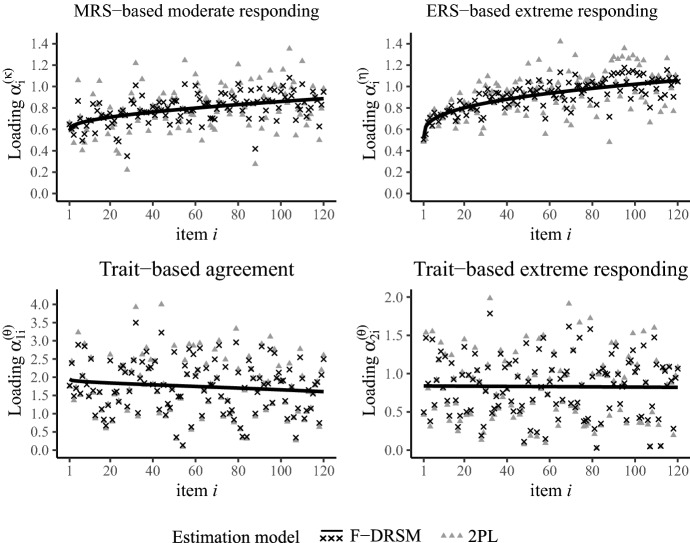
Loading estimates by the F-DRSM and 2PL model to the empirical data set.

For the analysis of dynamic response strategy changes, we examined the estimates of the DRSM, as the simulations suggested that it provides the most precise trajectory estimates. As hypothesized, the estimated slopes of MRS loadings $\alpha _{i}^{(\kappa )}$ and ERS loadings $\alpha _{i}^{(\eta )}$ were of substantial size and were both significantly larger than 0, meaning that the response strategy changed toward more RS involvement (see Table 4). Moreover, the influence of the substantive trait on fine-grained extreme decisions decreased, as indicated by a negative slope of loadings $\alpha _{2i}^{(\theta )}$, which is in line with the idea of RS-based processes taking over from trait-based responding in two-dimensional pseudo-items. This is original evidence that fine-grained decisions—and in particular, the RS-based processes—are highly dependent on the item position, suggesting an increase in fatigue and satisficing over the course of the questionnaire. Contrary to our assumption, we found that the trait loadings of the broad agreement decision ($\alpha _{1i}^{(\theta )}$) also decreased over time. However, as will be argued below, this is probably due to other item characteristics than the position within the questionnaire. Furthermore, the $\lambda$ estimates were 0.48 (MRS), 0.33 (ERS), 0.67 (trait-based agreement), and 0.59 (trait-based extreme responding), though only the 95 % CIs of the two RS trajectories did not include 1. This indicates that the response strategy change was most dominant in earlier item positions and is decelerated at the end of the questionnaire (also see Fig. 9).

The models were further compared by the LOO information criterion, which demonstrated that the F-DRSM provided the best fit (see Table 4). As was the case in the second simulation study, shrinking loadings to a dynamic trajectory was advantageous over freely estimating them or constraining them to a continuous function. The shrinkage effect is clearly visible in Fig. 9, which displays the estimated item-specific loadings by the 2PL model as well as the loadings and trajectories by the F-DRSM. Notably, the MRS and ERS loadings of the F-DRSM closely scatter around the respective estimated dynamic trajectories and the estimated *SDs* are 0.15 and 0.12, respectively. In contrast, the trait loadings of agreement and extreme responding scatter much stronger and the estimated *SDs* are 0.74 and 0.41, respectively.^4^ This indicates that the item position is a crucial determinant of the impact of RS-based response processes, whereas trait-based responding is greatly affected by other factors (e.g., other item characteristics or item content).

**Table 4:** Model fit and slope estimates for the empirical data set.

Model	LOO	Estimated slope and 95 %-CI			
		$\alpha _{i}^{(\kappa )}$	$\alpha _{i}^{(\eta )}$	$\alpha _{1i}^{(\theta )}$	$\alpha _{2i}^{(\theta )}$
Ordinal	311,417	0	0	0	0
ERS	305,271	0	0	0	0
Static	300,667	0	0	0	0
DRSM	300,433	0.31 [0.20, 0.42]	0.54 [0.43, 0.66]	$-$ 0.28 [$-$ 0.43, $-$ 0.16]	$-$ 0.12 [$-$ 0.22, $-$ 0.03]
F-DRSM	296,649	0.30 [0.14, 0.49]	0.54 [0.35, 0.72]	$-$ 0.34 [$-$ 0.92, 0.24]	$-$ 0.08 [$-$ 0.37, 0.28]
2PL	296,718	0.31 [0.13, 0.50]	0.57 [0.36, 0.79]	$-$ 0.38 [$-$ 1.02, 0.19]	$-$ 0.02 [$-$ 0.35, 0.31]

$\alpha _{i}^{(\kappa )}$ = loadings of MRS-based moderate responding; $\alpha _{i}^{(\eta )}$ = loadings of ERS-based extreme responding; $\alpha _{1i}^{(\theta )}$ = loadings of trait-based agreement; $\alpha _{2i}^{(\theta )}$ = loadings of trait-based extreme responding.

To give an example of how such additional factors can be investigated, we fitted an additional model with separate dynamic trajectories for positively and negatively worded items. For three of the four response processes, there were hardly any differences between the loading trajectories of positively and negatively worded items.^5^ Only trait-based agreement ($\alpha _{1i}^{(\theta )}$) differed largely between conditions: Positively worded items had high trait loadings throughout the questionnaire and the slope was not significantly different from zero ($\gamma _1 = 1.524$; $\gamma _I = 1.345$), whereas negatively worded items started with significantly smaller loadings, which then increased over items ($\gamma _1 = 0.990$; $\gamma _I = 1.265$). In the later part of the questionnaire, the conditions did not differ anymore (at 5 % error level). This suggests that it took the respondents some practice to process negatively worded items as accurately as positively worded ones. Further, negatively worded items occur more frequently in the later part of the IPIP-NEO-120, which would lead to artifacts such as the unexpected negative slope of trait-based agreement in the models not accounting for item wording (see $\alpha _{1i}^{(\theta )}$ in Table 4).

Taken together, our empirical application gave a first indication that there are qualitatively different mechanisms behind (1) response process of fine-grained decisions, which seem to be susceptible to response strategy changes toward heuristic responding (e.g., due to loss of motivation or fatigue) and (2) broad agreement decisions, which appear not to be affected by satisficing and reduced test-taking effort. However, more research focusing on response strategies as a psychological, cognitive construct is needed, in order to better understand how respondents arrive at their decisions, why they change their behavior over time, and how covariates (e.g., item wording) can explain different strategies.

## Discussion

The present research introduced the dynamic response strategy model (DRSM) as well as a more flexible extension (F-DRSM) and demonstrated how these models overcome the previous limitation of response style (RS) modeling, being that systematically changing influences of response processes over the items of a questionnaire were not accounted for. The new approaches address such dynamic response behaviors by modeling item position-dependent loadings of response processes (e.g., trait-based or RS-based response selection) in unidimensional and two-dimensional IRTree decision nodes. These loadings are assumed to follow continuous or non-continuous trajectories, which can either be dynamically changing with a linear or curvilinear shape, reflecting a response strategy change throughout the questionnaire, or be static ones, reflecting a constant response behavior. While the DRSM is an idealized and theory-driven model of strictly continuously changing loadings, the F-DRSM is adapted to more realistic, non-continuous settings, in which the item-specific loadings scatter around an underlying trajectory with normally distributed noise.

Simulation studies were conducted to compare the dynamic models and reasonable alternative models in terms of their suitability as cognitive explanatory models (i.e., requiring accurate quantification of dynamic changes of response processes and correct identification of non-dynamic, constant response strategies) as well as psychometric measurement models (i.e., requiring good recovery of person and item parameters and model fit). The DRSM turned out to be a capable cognitive model, as it reliably captured dynamically changing influences of response processes, irrespective of whether the data followed a continuous or non-continuous response strategy. Moreover, it did not pose the danger of misinterpretations due to falsely identified dynamics in the case of constant response strategies. Although perfectly continuous trajectories of response process loadings are most likely a simplification of real-world settings, the DRSM is a parsimonious formalization of the underlying general trend of the respondents’ behavior. Regardless of whether the model can capture all characteristics of a data set well, it proved to be a simple but appropriate model for investigating response strategies and changes of those over items. As such, it can be used to investigate how respondents arrive at their decisions, to uncover increasing satisficing or reduced test-taking effort, to evaluate questionnaires with regard to their cognitive load and burden, and to compare subgroups of respondents or items. The empirical application demonstrated that it is worthwhile to address such research questions, as the findings provided new insights on factors influencing the impact of different response processes on response selection, which eventually affect the data quality. Knowing about such factors, like item wording, can inform and improve test construction. Furthermore, constraining loadings to a trajectory is easy to implement, also outside Bayesian estimation methods, and requires only minor adjustments of the traditionally applied IRTree models and only few additional parameters to estimate. Therefore, the DRSM is a convenient model in applied fields of research to analyze personal characteristics, attitudes, or beliefs, and to control substantive trait estimates for dynamic RS effect.

Nevertheless, under realistic conditions of non-continuous response strategy changes, the constraint of continuous trajectories inevitably impairs the accurate representation of the true item-specific response behavior. Accordingly, the simulations showed that the more flexible models with item-specific influences of response process were superior in terms of psychometric properties. Notably, the F-DRSM with the underlying assumption of the response strategy being a composition of a systematic item position-dependent component and unsystematic noise was preferable not only to the continuous DRSM, but also to the unconstrained estimation of loadings by the 2PL model; both in simulated data and in the empirical application. Whenever the influences of response processes follow some sort of systematic over items, the F-DRSM effectively uses the information on this general trend for the estimation, making it a successful analysis model with good psychometric properties. The F-DRSM additionally comes with the advantage that the extent of unsystematic variance is estimated by the model so that the relative importance of the item position for the item-specific response strategy can be determined. For example, the empirical application not only showed that the influences of different response processes changed to different degrees over items, but also that the unsystematic variance greatly differed between trait-based and RS-based responding. The F-DRSM, therefore, allows to examine response strategies beyond the effect of the item position and opens up new possibilities for investigating the roles of different response processes in item responding. Overall, the two new dynamic models provide a surplus value to previous RS modeling because they facilitate the investigation of systematic heterogeneity of response processes across items of a questionnaire, which goes beyond existing approaches on discrete heterogeneity across measurement situations (e.g., Ames & Leventhal, 2021; Colombi et al., 2021; Weijters et al., 2010) or classes of respondents (e.g., Gollwitzer et al., 2005; Khorramdel et al., 2019; Meiser & Machunsky, 2008; Tijmstra et al., 2018). Thereby, our proposed models are a valuable first step toward transforming the heterogeneity of response processes over items into systematic variance, and toward answering the question of how respondents arrive at their judgments and decisions.

### Limitations and Future Directions

A limitation of the proposed models is that we defined the loading trajectories at the group level, which was based on the assumption that the factors leading to a change in the response strategy across items (e.g., loss of motivation) apply to all respondents to a similar extent. The estimated loading trajectories, however, can only represent the average response behavior and do not allow a differentiated assessment of individual response processes. In contrast, the mixture IRTree model by Kim and Bolt (2021) facilitates to distinguish groups of respondents by their response strategy, though under the strict assumption that all decisions during response selection are unidimensional and based either on the substantive trait or on the ERS, without considering a combination of processes, and without accommodating systematic changes. An integration of both approaches in the sense of a mixture DRSM (or F-DRSM) seems promising, as it may allow identifying classes of respondents with a dynamically changing response strategy, with a constant response strategy, and also respondents with only one of the two processes involved (which then follow the ERS or ordinal model). Such a model could be used in future studies to examine the heterogeneity of response processes between persons and simultaneously account for within-person changes across the items of a questionnaire.

A limitation of the conducted simulation studies is the chosen range of dynamic scenarios, which only partially covers all conceivable empirical response strategy changes. For instance, the simulated variation of the unsystematic component of the F-DRSM was lower than those we found for some processes in the empirical application. However, the process loadings in the simulation were centered around 0.5, while they were substantially larger in the application, which could naturally lead to a higher variance of the noise. Nonetheless, the conclusions drawn from the simulation study are limited to small or medium unsystematic variation, while the suitability of the F-DRSM for larger variations of loadings was suggested by empirical findings.

Further investigations might also be needed with regard to the shape of loading trajectories, as the proposed models are based on monotonous functions of loadings. If the response strategy changes were non-monotonic, for example, because respondents need to warm up to the task in the first part of a questionnaire, but lose motivation later on, U-shaped functions might be more appropriate. Even more complex loading patterns could result if respondents repeatedly alternate between responding with high and low effort, or if they start changing their strategy at a certain point within the questionnaire rather than from the very first item. Such complex patterns could in principle be investigated using the DRSM by replacing the curvilinear trajectories with other functions that match those hypotheses, although this would be challenging for model estimation and would likely require much larger data sets to achieve satisfactory precision of the results.

Also, the loadings might not only be associated with the time respondents spent on the test, which is captured by the item position, but further be dependent on the item content. For instance, in multidimensional questionnaires (such as the IPIP-NEO-120 used in the empirical application), the respondents’ interest or expertise might differ between trait dimensions, leading to a correlation of response process loadings with the measured dimensions. Though we have not addressed such dependencies, they could be empirically tested and accounted for by estimating dimension-specific trajectories, or by explaining the unsystematic variation of loadings in the F-DRSM by trait dimensions.

In addition, the present research investigated dynamic response strategies exclusively in the framework of IRTree models. However, our modeling approach with focus on response process loadings could likewise be integrated in other IRT model classes for which extensions for response styles were developed, such as the generalized partial credit model (Muraki, 1992) or the graded response model (Samejima, 1969). Notwithstanding such conceivable extensions, with the DRSM and F-DRSM, we have presented two of several ways to approach dynamic response processes in item responding, and leave further adaptations, developments, and generalizations to future research.

### Supplementary Information

Below is the link to the electronic supplementary material.

Supplementary file 1 (pdf 437 KB)

## Footnotes

1 We thank an anonymous reviewer for pointing out the importance of distinguishing between positive and negative response process loadings.

2 The center of all trait and ERS trajectories, that is $(\gamma _{1} + \gamma _{I})/2$, was set to 0.5. This value was chosen for both response processes because a prior study (Meiser et al., 2019) and our empirical application indicated (1) that trait-based and RS-based processes have about the same influence on two-dimensional IRTree decisions, and (2) that the loadings of the substantive trait are roughly twice as large in the broad agreement decision compared to fine-grained response selection (e.g., in extreme decision nodes).

3 The detailed results of all replications conducted in the simulation study can be found on the Open Science Framework repository: https://osf.io/kc8ve/.

4 The second simulation study revealed accurate recovery of the *SD* of the unsystematic component. Across replications with low *SD* of 0.1: $M = 0.103, SD = 0.023$; with high *SD* of 0.2: $M = 0.201, SD = 0.030$.

5 The only significant differences at 5 % error level were in $\gamma _I$ and slope of the trait loadings of extreme responding ($\alpha _{2i}^{(\theta )}$), which indicated that loadings of positively worded items had a stronger decline.
