# Supplementary material for: Dynamic Response Strategies: Accounting for Response Process Heterogeneity in IRTree Decision Nodes
Source: Psychometrika. 2023 Feb 6;88(4):1354–80. doi: 10.1007/s11336-023-09901-0 (PMC10656330; doi:10.1007/s11336-023-09901-0)
Supplement: Supplementary file 1 — (pdf 437 KB) [file 11336_2023_9901_MOESM1_ESM.pdf]

Table A1.
Posterior *SDs* of DRSM trajectory parameters in simulation study 1.

			Posterior *SDs*	
Parameter	*N*	*I*	*M*	*SD*
γ_1	500	20	0.085	0.018
		40	0.069	0.018
	1000	20	0.067	0.015
		40	0.048	0.015
γ_1	500	20	0.079	0.018
		40	0.051	0.012
	1000	20	0.053	0.013
		40	0.038	0.009
λ	500	20	0.705	0.177
		40	0.578	0.232
	1000	20	0.582	0.232
		40	0.523	0.232

Table A2.
Estimated slopes by the DRSM fitted to non-dynamic data in simulation study 1.

Data generation		Estimated slope by DRSM	
Model	Process	*M*	*SD*
Ordinal	Trait	0.003	0.093
	ERS	-0.014	0.061
ERS	Trait	-0.005	0.025
	ERS	0.007	0.096
Static	Trait	0.002	0.088
	ERS	0.006	0.107

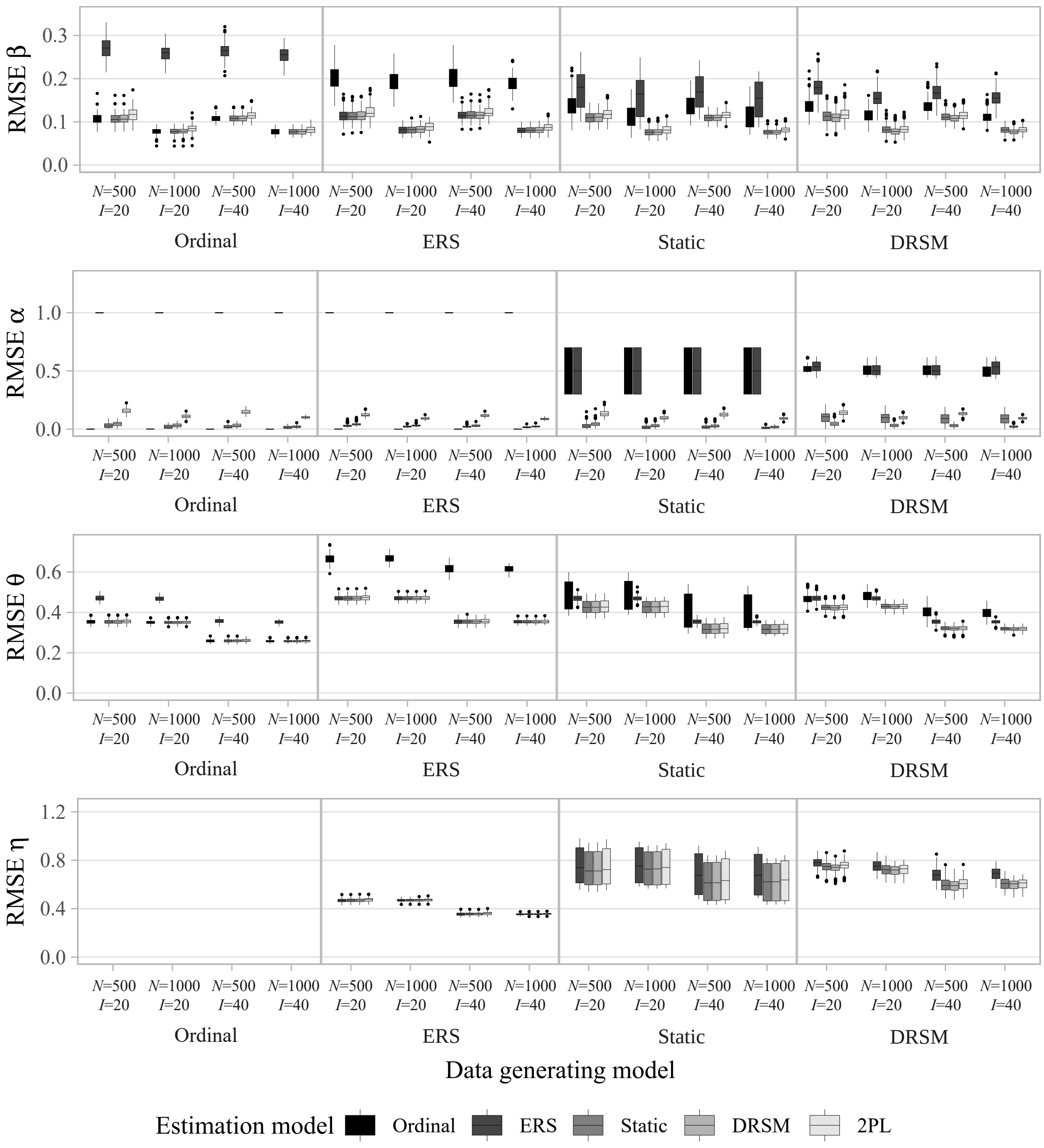

FIGURE A1.
RMSEs of estimated person and item parameters for continuous data in simulation study 1.

Table A3.
Comparison of estimated slopes by DRSM, F-DRSM and 2PL model for non-continuous data in simulation study 2.

Data generation		Estimated absolute slope M (SD)			Mean posterior SD				
Abs. slope	SD	DRSM	F-DRSM	2PL	DRSM	F-DRSM	2PL	Min. cor.	MAD
0.0	0.1	-0.004 (0.094)	-0.005 (0.090)	-0.007 (0.093)	0.061	0.091	0.095	0.971	0.009
	0.2	0.007 (0.170)	0.005 (0.150)	0.005 (0.152)	0.065	0.147	0.150	0.968	0.029
0.2	0.1	0.215 (0.083)	0.212 (0.082)	0.215 (0.086)	0.060	0.090	0.094	0.965	0.010
	0.2	0.216 (0.133)	0.203 (0.128)	0.206 (0.130)	0.062	0.144	0.147	0.954	0.027
0.4	0.1	0.415 (0.084)	0.415 (0.083)	0.422 (0.084)	0.059	0.088	0.090	0.969	0.011
	0.2	0.414 (0.133)	0.408 (0.131)	0.413 (0.132)	0.059	0.133	0.135	0.954	0.027
0.6	0.1	0.609 (0.078)	0.609 (0.075)	0.615 (0.077)	0.058	0.084	0.084	0.967	0.010
	0.2	0.608 (0.127)	0.602 (0.124)	0.606 (0.127)	0.059	0.129	0.130	0.955	0.026

Min. cor. = minimum correlation of slope estimates across the three models; MAD = average of pairwise calculated mean absolute difference of slope estimates across the three models.
